# Study of Cellulose Dissolution in ZnO/NaOH/Water Solvent Solution and Its Temperature-Dependent Effect Using Molecular Dynamics Simulation

**DOI:** 10.3390/polym16091211

**Published:** 2024-04-26

**Authors:** Lamiae Bourassi, Meriem El Mrani, Mohammed Merzouki, Rania Abidi, Haytham Bouammali, Boufelja Bouammali, Larbi Elfarh, Rachid Touzani, Allal Challioui, Mohamed Siaj

**Affiliations:** 1 Laboratory of Applied Chemistry and Environment (LCAE), Organic Macromolecular Chemistry & Phytochemistry (ECOMP), Faculty of Sciences, Mohammed First University, Oujda 62000, Morocco; lamiae.bourassi@ump.ac.ma (L.B.); meriemelmrani2000@gmail.com (M.E.M.); moh.merzouki@gmail.com (M.M.); rania.abidi@ump.ac.ma (R.A.); bouammalihaytham@gmail.com (H.B.); a_bouammali@yahoo.fr (B.B.); allal.challioui@gmail.com (A.C.); 2 Laboratory of Theoretical Physics, Particles, Modeling and Energies (LPTPME), Faculty of Sciences, Mohammed First University, Oujda 62000, Morocco; 3 Chemistry Department, Université Québec A Montréal, Montréal, QC H3C 3P8, Canada; siaj.mohamed@uqam.ca

**Keywords:** cellulose, MD simulation, cellulose dissolution, ZnO/NaOH aqueous solution, hydrogen bonding, temperature behavior of cellulose, cellulose, the interaction energy between cellulose and aqueous solvent

## Abstract

Cellulose is a biopolymer with numerous advantages that make it an ecological, economical, and high-performing choice for various applications. To fully exploit the potential of cellulose, it is often necessary to dissolve it, which poses a current challenge. The aqueous zinc oxide/sodium hydroxide (ZnO/NaOH/Water) system is a preferred solvent for its rapid dissolution, non-toxicity, low cost, and environmentally friendly nature. In this context, the behavior of cellulose chains in the aqueous solution of ZnO/NaOH and the impact of temperature on the solubility of this polymer were examined through a molecular dynamics simulation. The analysis of the root means square deviation (RMSD), interaction energy, hydrogen bond curves, and radial distribution function revealed that cellulose is insoluble in the ZnO/NaOH solvent at room temperature (T = 298 K). Decreasing the temperature in the range of 273 K to 268 K led to a geometric deformation of cellulose chains, accompanied by a decrease in the number of interchain hydrogen bonds over the simulation time, thus confirming the solubility of cellulose in this system between T = 273 K and T = 268 K.

## 1. Introduction

**Figure 1:**
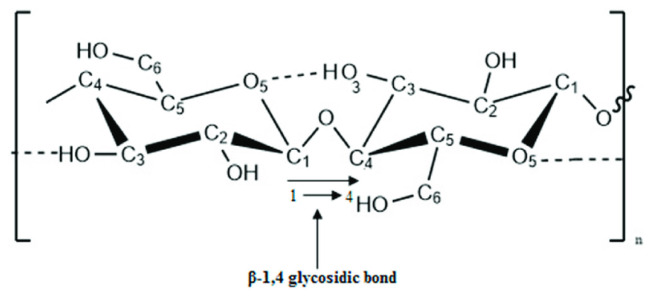
Structure of cellulose.

Growing concern over environmental pollution has prompted researchers to shift their attention from traditional petroleum-derived synthetic polymers to more environmentally friendly alternatives [1]. Among the latter, cellulose, the most abundant biopolymer on Earth, stands out as an available renewable organic material, offering advantages such as its non-toxicity, broad durability, and environmental friendliness [2,3]. Additionally, cellulose has been used for thousands of years in the manufacture of fibers, paper, films, filters, and textiles [4], making it a promising option to meet today’s needs for environmentally friendly materials [5,6]. Cellulose is a syndiotactic biopolymer of anhydroglucose units (AGUs) linked by (1,4) β-glycosidic bonds (Figure 1) [3].

The fundamental characteristic that gives cellulose its unique properties is its dynamic crystallinity and its ability to arrange in different crystalline forms, known as polymorphs [3]. The crystallinity of cellulose refers to the way in which the cellulose chains are organized in a solid structure with a high degree of regularity and orderliness [7]. This crystalline organization gives cellulose distinctive physical properties, such as mechanical strength, rigidity, dimensional stability, and insolubility, in many solvents [8].

In the crystalline regions, the cellulose chains are joined together by complex intra- and intermolecular hydrogen bonds, forming the basis of cohesion between the cellulose chains. In contrast, in the amorphous domains, the cellulose chains are distributed homogeneously along the microfibers [9,10]. The intra- and interchain hydrogen bonds give the cellulose fiber a stable and compact structure, while the network of hydrogen bonds is one of the main factors of its insolubility in organic and inorganic solvents and its resistance to decomposition by microbial and enzymatic agents [1,11]. The solubility of cellulose in alkaline aqueous systems (MOH where M = Na, K) is closely related to the reactivity of the hydroxyl groups, particularly the C6-OH, within its structure. Indeed, the -C6-OH group is highly reactive [12], and its availability for nucleophilic reactions is significantly influenced by the presence of hydroxide ions (OH^−^). When cellulose is exposed to alkaline conditions, such as those used in pretreatment procedures [13], the reactivity of C6-OH is further enhanced, thus facilitating cellulose dissolution. Therefore, understanding in detail how hydrogen bonds involving C6-OH form and break, whether within the same cellulose molecule or between different molecules, is essential for elucidating the mechanisms of cellulose dissolution. This knowledge will enable the design of more effective treatment methods to enhance the solubility of cellulose in various solvents, which is crucial for the development of new cellulose-derived materials with improved properties. Another factor contributing to the insolubility of cellulose is its amphiphilic nature, which refers to the presence of both hydrophilic (water-attracting) and hydrophobic (water-repelling) functional groups in its molecular structure [14]. Thus, cellulose is made up of long glucose chains, and each glucose unit contains three hydroxyl groups (-OH) located on the equatorial positions of the glucose ring. These hydroxyl groups are hydrophilic with a strong affinity for water through hydrogen bonds. However, cellulose also has hydrophobic CH bonds (carbon–hydrogen bonds) located on the axial positions of the glucose ring and is, therefore, less interactive with water [15,16]. Therefore, the disturbance in the balance between the hydrophilic and hydrophobic character can play a crucial role in its dissolution [13,17]. Cellulose is also renowned for its resistance to dissolution in many organic and inorganic solvents, making it essentially insoluble in water, acetone, ethanol, and many other liquids [18]. This insolubility is essential to its structural role in plants, but it also raises fundamental questions and provides exciting opportunities for research and innovation.

There are several factors that influence the solubility of cellulose, including cellulose particle size and its degree of polymerization and molecular weight. Indeed, smaller cellulose particles tend to be more soluble than larger ones, such as cellulose fibers obtained from wood pulp, cotton, or other natural sources, which have reduced solubility in water and in many organic solvents. This reduced solubility is mainly due to crystalline regions of the cellulose fibers and the hydrogen bonds. The latter creates a network that resists the penetration of solvent molecules and inhibits dissolution [19,20]. On the other hand, the degree of polymerization and molecular weight significantly influence its solubility. Thus, a degree of polymerization (DP) between 100 and 20,000 is insoluble in water and in a large part of organic and inorganic solvents, while shorter fragments of cellulose, with a DP ranging from 2 to around 12, are generally soluble [21].

Other factors, such as temperature, pressure, and solvent concentration, can influence the solubility of cellulose. In addition, the aqueous sodium hydroxide solution is a preferred solvent for cellulose dissolution due to its various advantages, including non-toxicity, low cost, environmentally friendly, and fast dissolution process [22,23,24]. The main limitations lie in the low concentration of cellulose that can be dissolved in the aqueous NaOH solution (less than 10%) [25], as well as in the gelation that occurs over time and with increasing temperature. This is why several dissolution processes of cellulose include the addition of organic and/or inorganic moieties such as zinc oxide (ZnO), urea, and thiourea to improve the performance of the alkaline aqueous solutions. In particular, ZnO is known as an additive in the alkali dissolution in NaOH/aqueous system, which allows the formation of strong hydrogen bonds between cellulose and Zn (OH)_4_^2−^ anion, leading to cellulose dissolution [4,26,27]. The latter can also enhance cellulose dissolution in the aqueous solvent ZnO/NaOH by associating and charging cellulose molecules under alkaline conditions [4]. In addition, it has been suggested that ZnO acts as a ‘binder’ for water, stabilizing cellulose solutions in aqueous NaOH through additional interactions between water molecules and ${Zn\left(OH\right)}_{3}^{-}$ and ${Zn\left(OH\right)}_{4}^{2-}$ ions. These interactions significantly reduce the amount of free water around cellulose chains, thereby reducing its aggregation. Therefore, ZnO stabilizes the solution by keeping water away from the cellulose chains [28]. On the other hand, temperature is a very important parameter controlling the cellulose dissolution in NaOH-based solutions, and unlike the general idea that entropy plays a greater role when increasing temperature, it has been found that decreasing temperatures below zero is generally required to dissolve cellulose in this type of solutions [2,29].

Recently, Bregado et al. [30] observed that temperature influences the dissolution of a crystalline model of cellulose Iβ microfibrils at 25 MPa in the temperature range of 298–660 K. Similarly, Ramakrishnan et al. [31] showed that the dissolution of cellulose in a mixture of 1-ethyl-3-methylimidazolium acetate ionic liquid [C2C1Im][OAc] with water is temperature dependent. By exploring the evolution of the RMSD parameters and the number of H-bonds in molecular dynamics studies, the authors confirmed that T = 433 K is an effective temperature for dissolving cellulose in this solvent system. 

In this study, we investigate the mechanism of cellulose solubility in an aqueous ZnO/NaOH system at different temperatures by using molecular dynamics (MD) simulation. The aim is to draw a clear scheme of temperature-dependent conformational changes in the cellulose chains, which is generally characterized by the disruption of the interchain hydrogen bond network at low temperatures, leading to the separation of tightly packed chains from the crystalline structure and the dissolution of the cellulose.

## 2. Materials and Methods

The molecular dynamics of cellulose solubility at different temperatures were simulated using Schrödinger suite software (2018.4). The cellulose structure is an Iβ crystalline structure containing 6 chains with 8 glucose units, constructed using cellulose builder [32]. The force field used is OPLS-2005 [33]. The size of the periodic simulation box was 10 × 10 × 10 Å. The solvent system consisted of water molecules modeled using the TIP3P model, ZnO, Na^+^, and OH^−^ molecules, composed of 9% NaOH/1% ZnO modeled using the Material package integrated into the Schrödinger 2018.4 software with the same force field. The simulations were performed in a time of 10 ns with an interval of 2 fs under a canonical ensemble (NVT) with the pressure controlled by a Berendsen barostat P = 1 bar, and each simulation was performed at a different temperature; the temperatures studied are 260, 273, 278, 290, and 298. The simulations were carried out using the Desmond package 2018.4.

## 3. Results and Discussion

### 3.1. Root Mean Square Deviation (RMSD)

**Figure 2:**
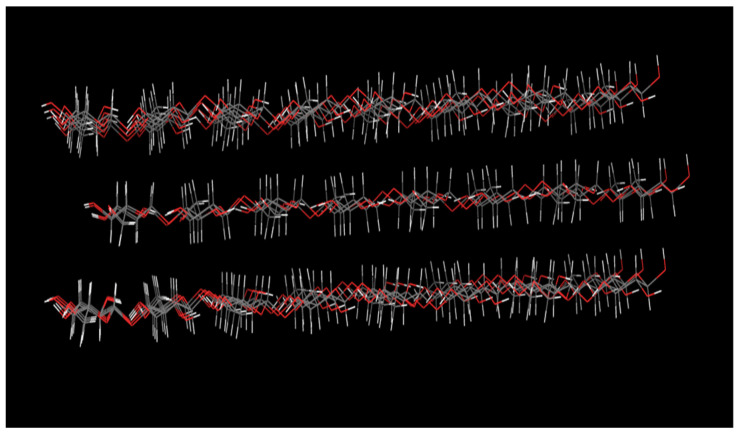
Images capturing the cellulose structures at 0 ns.

**Figure 3:**
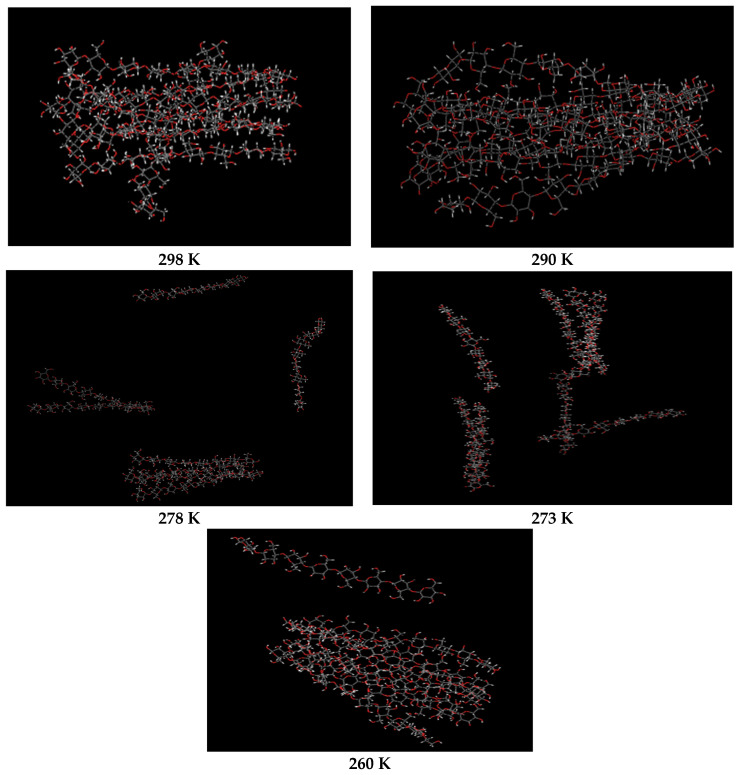
Images capturing the cellulose structures at 10 ns at different temperatures.

To control the process of cellulose dissolution in the aqueous ZnO/NaOH solvent, an approach was adopted to study the evolution of chain conformation during the MD simulation. This analysis aims to understand the dissolution mechanisms and to identify the key factors influencing cellulose solubility by following the evolution of cellulose chains at different times during the simulation (i.e., the initial time (0 ns) (Figure 2) and at the final time (10 ns) (Figure 3), under different temperature conditions (i.e., T = 260 K, 273 K, 278 K, 290 K, and 298 K).

The visual representations in the final simulation times in (Figure 3) show that the cellulose chains are in an aggregated form at temperatures T = 260 K, 290 K, and 298 K (Figure 3). However, a different behavior is observed at T = 273 K and 278 K, where the cellulose chains adopt a side-by-side ordered configuration at the beginning of the simulation at t = 0 ns (Figure 2). However, a continuous configurational arrangement of the chains is observed during the simulation, transitioning from a regular linear form to a deformed form, leading to a complete separation of the chains at the end of the simulation at 10 ns (Figure 3). This segregation of chains indicates the dispersion of the cellulose chains in the medium, suggesting that the dissolution of cellulose in an aqueous ZnO/NaOH solvent system occurs at these temperatures. These findings are supported by experimental work conducted by Kang et al. [34], who investigated the dissolution and molecular interactions of cellulose carbamate (CC) in NaOH/ZnO aqueous solutions. Their analyses, utilizing various techniques such as optical microscopy, differential scanning calorimetry (DSC), proton nuclear magnetic resonance (1H NMR), dynamic light scattering (DLS), atomic force microscopy (AFM), and transmission electron microscopy (TEM), confirmed that lower temperatures were favorable for CC dissolution in the ZnO/NaOH system. Another experimental study conducted by Väisänen et al. [4] on the dissolution of cellulose in a ZnO/NaOH solvent system was monitored using Raman spectroscopy. The authors showed that the dissolution of cellulose in aqueous NaOH at low temperatures is generally attributed to the formation of NaOH hydrates capable of penetrating the cellulose network, thereby detaching individual chains from each other and forming a new network with them. Additionally, the addition of ZnO facilitates the dissolution of cellulose and delays its self-aggregation by coordinately binding to the C2 and C3 OH groups of cellulose chains released from the crystalline structure, thus forming a ring-like structure similar to zinc glycerolate. The formation of this complex disturbs the conjugated electronic system of the hydrogenated cellulose network and thereby helps keep the cellulose chains apart from each other.

To better understand the solubility of cellulose at different temperatures, we analyzed the root mean square deviation (RMSD) as a key parameter to monitor the evolution of structures during simulations and observe the dispersion of cellulose chains in the solvent system. The RMSD provides a measure of the average deviation between the atoms of a molecule relative to a reference structure over time. Indeed, it quantifies the fluctuations in the position of atoms within a molecule relative to a reference structure over time. Thus, for cellulose dissolved in ZnO/NaOH, a stable RMSD implies that the cellulose structure remains relatively unchanged, with few notable modifications in its spatial configuration during the observed period. Cellulose insolubility refers to its resistance to dissolve, or to partially dissolve, in the ZnO/NaOH solvent. In other words, low cellulose solubility may be associated with high RMSD stability, suggesting that the cellulose structure remains intact and is not sufficiently disturbed to allow complete dissolution in the solvent, while a variation in RMSD indicates the perturbation of the cohesion of the arrangement of the cellulose chains. This is why the correlation between RMSD and cellulose solubility is frequently investigated in molecular dynamics simulations [30,31]. In our study, we calculated the average RMSD of the cellulose chains relative to the ZnO/NaOH solvent at different temperatures to explore how temperature variations affect the stability and dissolution of cellulose.

**Figure 4:**
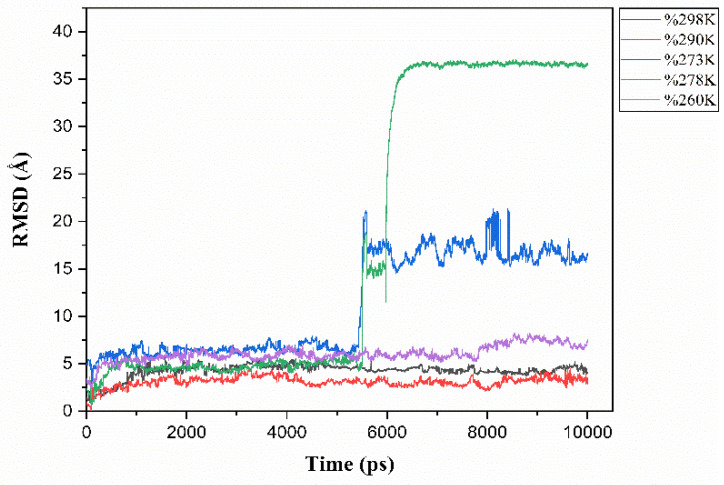
RMSD values of cellulose in ZnO/NaOH aqueous solvent at different temperatures.

Thus, by calculating the root mean square deviation (RMSD) of cellulose chains for each temperature (Figure 4) and comparing the RMSD curves, we were able to follow the evolution of cellulose structures under different thermal conditions, whereas by monitoring the RMSD fluctuations, we could estimate the effectiveness of cellulose solubility at each temperature since stability in RMSD values indicates insolubility [35]. Thus, at temperatures of 273 K and 278 K, the results show an increase in RMSD values over the simulation period, suggesting deformation in the crystalline structure of cellulose and, consequently, its solubility. This temperature range is consistent with other studies, as indicated in the work of Zhang et al., who reported that cellulose solubility in the NaOH/urea system is observed between −10 °C and 5 °C, while a frozen phase appears below −10 °C [21].

### 3.2. Interaction Energy

**Figure 5:**
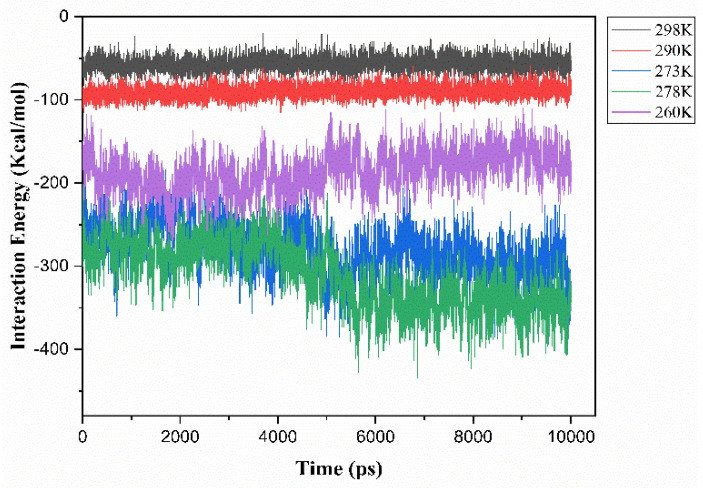
Interaction energy of cellulose in ZnO/NaOH aqueous solvent at different temperatures.

The dissolution of cellulose involves breaking its interchain bonded state into individual chains. The strength of these bonds, represented by the interaction energy between cellulose chains, affects their packing density. To study the effect of temperature on cellulose dissolution in the ZnO/NaOH aqueous solvent system, we analyzed the average electrostatic interaction energy between cellulose chains and the solvent at various temperatures using the Schrödinger Suite software (version 2018.4) and the Desmond package. The results (Figure 5) show that at temperatures close to room temperature (298–290 K) and below 260 K, no significant variation is observed in the interaction energy curves. However, a decrease in interaction energy is noted when the temperature decreases to a range between 278 K and 273 K and suggests a stronger preference for interaction between cellulose chains and the solvent. As the temperature increases or decreases, the interaction between cellulose and the solvent becomes less favorable due to a strengthening of the bonding forces between cellulose chains that hinder their dissolution in the solvent. This trend in cellulose–solvent interaction agrees with the RMSD analysis, indicating stronger interactions between cellulose and the solvent at 278 K–273 K compared to lower and higher temperatures.

### 3.3. Hydrogen Bonds

**Figure 6:**
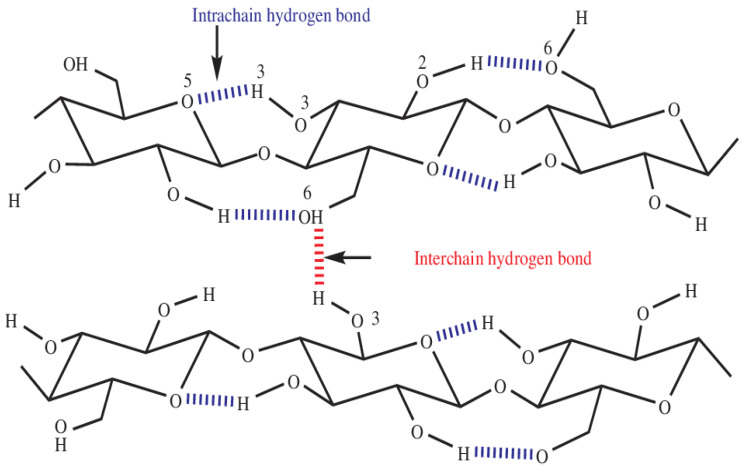
Intra-interchain hydrogen bonds of cellulose [36].

Hydrogen bonds are of primordial importance in the close packing and the stabilization of crystalline Iβ cellulose, which exhibits two types of hydrogen bonds: intramolecular bonds within cellulose chains and intermolecular bonds between chains (Figure 6). It is, therefore, possible to monitor variations in hydrogen bonds within chains and between chains to analyze the dissolution of cellulose in various solvent systems. This analysis enables us to understand the mechanisms underlying dissolution and optimize cellulose processing conditions.

**Figure 7:**
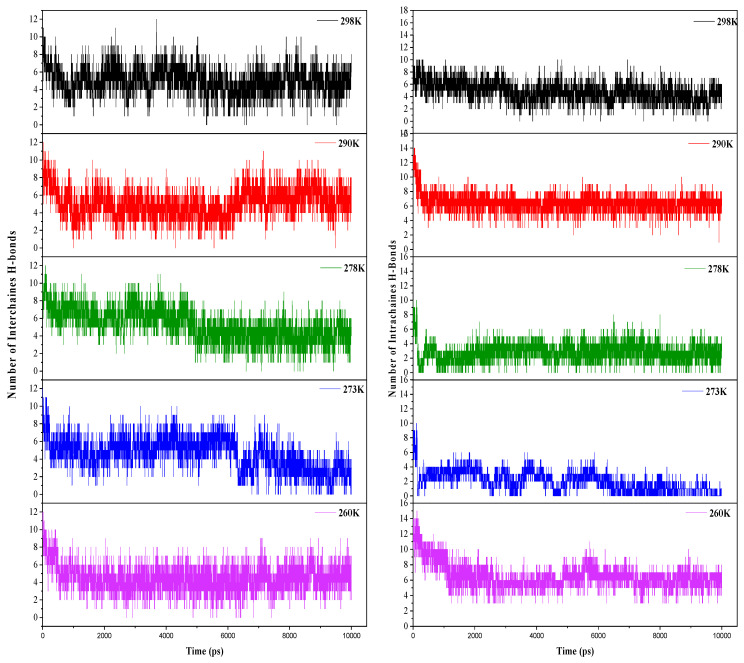
Analyses of inter-intrachain H bonds at different temperatures.

When analyzing the evolution of hydrogen bonds vs temperature, we observe that within the temperature range of 290 K to 298 K, a relatively stable number of inter- and intrachain hydrogen bonds (Figure 7) indicates the stability of the cellulose structure. However, significant variations emerge at intermediate temperatures, specifically between 278 K and 273 K during simulations (Figure 7). The number of intrachain hydrogen bonds decreases from 10 to 3, while interchain bonds drop from 12 to 7. This pronounced decrease at intermediate temperatures suggests a notable alteration in the molecular structure of cellulose due to specific interactions of cellulose chains with the ZnO/NaOH aqueous system. These observations raise the hypothesis of increased disruption of inter- and intrachain hydrogen bonds at lower temperatures, potentially leading to greater accessibility of hydroxyl groups to interact with the solvent. Therefore, temperature plays a crucial role in the dynamics of hydrogen bonds and, consequently, in the controlled dissolution of cellulose within this system. At 260 K (Figure 7), stability in the number of hydrogen bonds is once again observed, indicating a possible saturation of molecular interactions at this lower temperature.

**Figure 8:**
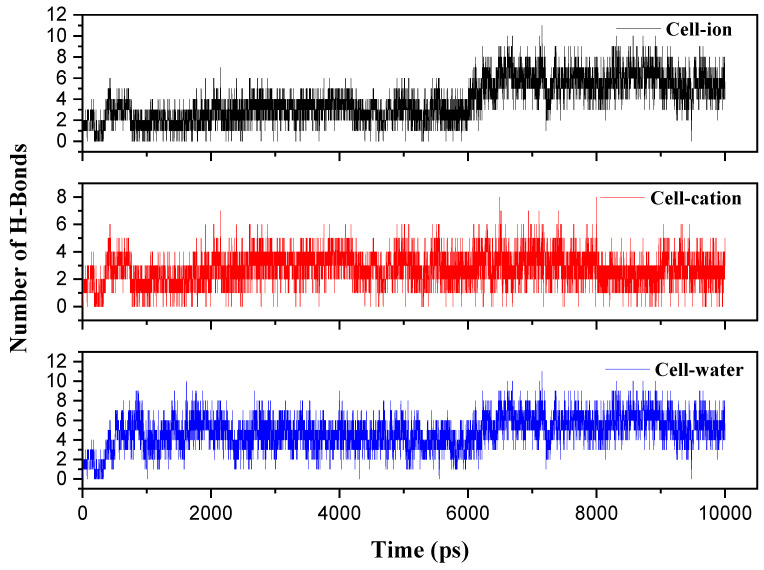
Graph showing the average number of hydrogen bonds between cellulose and cations (red), cellulose and anions (black), and cellulose and water (blue), evaluated at T = 278 K.

To confirm the accessibility of the cellulose hydroxyl groups to solvent molecules after the breaking of intra- and interchain bonds of cellulose, we chose to measure the number of hydrogen bonds between cellulose and anions, cations, and water throughout the simulation. It is well established, according to Liu et al. [37], that during the cellulose dissolution process, the hydroxyl groups of the AGU unit can form hydrogen bonds with the anions, significantly influencing cellulose–solvent interactions. The results of hydrogen bonds between cellulose and solvent at T = 278 K show a significant increase in the number of hydrogen bonds. Specifically, the numbers are higher between cellulose and anions, ranging from 8 to 14 (Figure 8), and between cellulose and water, the number of bonds slightly increases from 7 to 10 (Figure 8) compared to bonds between cellulose and cations. These results confirm that at a temperature of 278 K, the hydroxyl groups of the glucose unit can form hydrogen bonds with the solvent. Additionally, the hydroxyl groups of cellulose form a higher number of bonds with the anions compared to water, suggesting that cellulose is more soluble with the addition of ZnO/NaOH in the aqueous system at a temperature of 278 K.

### 3.4. Radial Distribution Function (RDF)

The radial distribution function (RDF) is a crucial parameter in molecular dynamics simulations, revealing the specific solute–solvent distribution in different systems. This distribution provides a better understanding of interactions on a molecular scale. Specifically, RDF describes how the density of a given particle varies as a function of distance from a reference particle [33,38]. In our case, we chose the three hydroxyl groups (O_2_, O_3_, and O_6_) in the glucose units, which are responsible for hydrogen bonding, to calculate the RDF between cellulose and other solvent species, including Zn(OH^−^)_4_ molecules, Na^+^ ions, and water molecules.

The radial distribution function is represented by the following equation [39]:

$$g_{ij}\left(r\right)=\frac{N_{ij}\left(r,r+∆r\right)V}{4\pi r^{2}∆rN_{i}j}$$

where r is the distance between atom *i* and *j*, $N_{ij}\left(r,r+∆r\right)V$ is the number of j particles around *i* within a shell radius of r normalized by the actual number of $N_{i}$ and $N_{j}$ atoms at that distance, and V is the total volume of the system.

**Figure 9:**
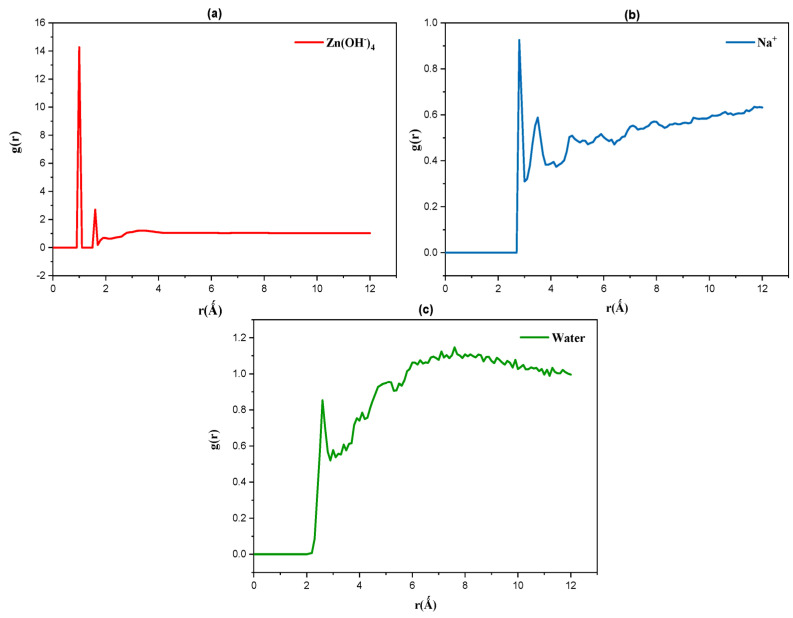
RDF curves between cellulose and the various solvent components. (**a**) cellulose-cation, (**b**) cellulose-water, (**c**) cellulose-anion.

Based on the previous analyses, the most interesting temperature is T = 273 K. Therefore, in this study, we analyzed the RDF curves between cellulose and the various solvent components at T = 273 K (Figure 9a–c).

For the interaction between cellulose and the cation (Figure 9a), the curve reveals peaks corresponding to the first interaction shell of cellulose–cation at distances of r = 2.8 Å and 3.3 Å. Between cellulose and water (Figure 9b), the first peak with low intensity is observed at a distance of r = 2.1 Å. Conversely, between cellulose and the anion (Figure 9c), the distances show smaller values: r = 1 Å and 1.3 Å. The coordination numbers (CN) of the cellulose–solvent interaction obtained by integrating the RDF results up to the first minimum (3.35 Å) show that between cellulose and cations, the values are between 0.03 and 0.04. For the interaction between cellulose and water, the CN ranges between 0.05 and 0.07, while for the interaction between cellulose and the anion, the CN ranges between 1.3 and 2.2.

Comparing the distances between cellulose–anion, cellulose–cation, and cellulose–water, it is evident that the anion is situated at a shorter distance compared to the cation and water, with interaction peaks also being more intense than those observed for the cation and water. This suggests that the anion presents a denser distribution around the cellulose hydroxyl groups in the first solvation shell. Conversely, the cation and water are situated at greater distances, and the likelihood of water distribution around the cellulose hydroxyl groups is higher than that of the cation.

These findings are supported by the CN values between cellulose and the cation, which indicate that the distribution of cations around the cellulose hydroxyl groups is highly limited, resulting in weak cellulose–cation interaction. Based on RDF analysis, a spatial organization of solvating species around the cellulose hydroxyl groups is predicted in the ZnO/NaOH aqueous mixture at T = 278 K. In the first solvation shell, the cellulose hydroxyl groups are surrounded by Zn(OH^−^)_4_ molecules, followed by water molecules, and then by Na^+^ cations. This distribution could facilitate the dissolution process at this temperature in this system.

## 4. Conclusions

Molecular dynamics simulations were conducted to investigate the effect of temperature on cellulose dissolved in an aqueous ZnO/NaOH system. The simulations were performed at different temperatures: T = 298 K, 290 K, 278 K, 273 K, and 260 K. This simulation study provides a microscopic insight into the behavior of cellulose chains at different temperatures. At 298 K, 290 K, and 260 K, the chains exhibit some stability and remain primarily in an ordered form throughout the simulation. However, at lower temperatures, T = 278 K and 273 K, the chains completely separate, indicating dispersion of the cellulose chains in the medium. These results suggest that the dissolution of cellulose in an aqueous ZnO/NaOH solvent system occurs at temperatures between 273 K and 278 K. This conclusion is supported by the analysis of RMSD, which shows an increase in RMSD values at 278 K and 273 K. Additionally, the analysis of intra- and intermolecular hydrogen bonds indicates that these bonds decrease at T = 278 K–273 K, which may lead to greater accessibility of the hydroxyl groups to interact with the solvent. This increased accessibility would facilitate the dissolution process of cellulose in ZnO/NaOH at temperatures between 278 K and 273 K.

Regarding the mechanism of solvent interaction with cellulose, the results of radial distribution function (RDF) clarify that the Zn(OH^−^)_4_ anion is the most crucial solvent component in the cellulose dissolution process in the aqueous ZnO/NaOH system. Therefore, according to this study, cellulose dissolution in ZnO/NaOH is preferable at lower temperatures between 273 K and 278 K.

## Data Availability

Data are contained within the article.

## References

[1] Thongsomboon W., Serra D.O., Possling A., Hadjineophytou C., Hengge R., Cegelski L. (2018). Phosphoethanolamine Cellulose: A naturally Produced Chemically Modified Cellulose. Science.

[2] Wang Y., Liu L., Chen P., Zhang L., Lu A. (2018). Cationic hydrophobicity promotes dissolution of cellulose in aqueous basic solution by freezing-thawing. Phys. Chem. Chem. Phys..

[3] Heinze T. (2015). Cellulose: Structure and properties. Adv. Polym. Sci..

[4] Väisänen S., Ajdary R., Altgen M., Nieminen K., Kesari K.K., Ruokolainen J., Rojas O.J., Vuorinen T. (2021). Cellulose dissolution in aqueous NaOH–ZnO: Cellulose reactivity and the role of ZnO. Cellulose.

[5] Seddiqi H., Oliaei E., Honarkar H., Jin J., Geonzon L.C., Bacabac R.G., Klein-Nulend J. (2021). Cellulose and its derivatives: Towards biomedical applications. Cellulose.

[6] Fu L.H., Qi C., Ma M.G., Wan P. (2019). Multifunctional cellulose-based hydrogels for biomedical applications. J. Mater. Chem. B.

[7] Ruel K., Nishiyama Y., Joseleau J.P. (2012). Crystalline and amorphous cellulose in the secondary walls of Arabidopsis. Plant Sci..

[8] Emenike E.C., Iwuozor K.O., Saliu O.D., Ramontja J., Adeniyi A.G. (2023). Advances in the extraction, classification, modification, emerging and advanced applications of crystalline cellulose: A review. Carbohydr. Polym. Technol. Appl..

[9] Liu R., Yu H., Huang Y. (2005). Structure and morphology of cellulose in wheat straw. Cellulose.

[10] Poletto M., Pistor V., Zattera A.J. (2013). Structural Characteristics and Thermal Properties of Native Cellulose. Cellulose—Fundamental Aspects.

[11] Bochek A.M. (2003). Effect of Hydrogen Bonding on Cellulose Solubility in Aqueous and Nonaqueous Solvents. Russ. J. Appl. Chem..

[12] Heise K., Koso T., King A.W.T., Nypelö T., Penttilä P., Tardy B.L., Beaumont M. (2022). Spatioselective surface chemistry for the production of functional and chemically anisotropic nanocellulose colloids. J. Mater. Chem. A.

[13] Bali G., Meng X., Deneff J.I., Sun Q., Ragauskas A.J. (2015). The effect of alkaline pretreatment methods on cellulose structure and accessibility. ChemSusChem.

[14] Malaspina D.C., Faraudo J. (2019). Molecular insight into the wetting behavior and amphiphilic character of cellulose nanocrystals. Adv. Colloid Interface Sci..

[15] Biermann O., Hädicke E., Koltzenburg S., Müller-Plathe F., Müller-Plathe F. (2001). Hydrophilicity and Lipophilicity of Cellulose Crystal Surfaces. Angew. Chem. Int. Ed..

[16] Isogai A., Hänninen T., Fujisawa S., Saito T. (2018). Review: Catalytic oxidation of cellulose with nitroxyl radicals under aqueous conditions. Prog. Polym. Sci..

[17] Miyamoto H., Umemura M., Aoyagi T., Yamane C., Ueda K., Takahashi K. (2009). Structural reorganization of molecular sheets derived from cellulose II by molecular dynamics simulations. Carbohydr. Res..

[18] Medronho B., Romano A., Miguel M.G., Stigsson L., Lindman B. (2012). Rationalizing cellulose (in)solubility: Reviewing basic physicochemical aspects and role of hydrophobic interactions. Cellulose.

[19] Mohd N., Draman S.F.S., Salleh M.S.N., Yusof N.B. (2017). Dissolution of cellulose in ionic liquid: A review. AIP Conf. Proc..

[20] Singh P., Duarte H., Alves L., Antunes F., Le Moigne N., Dormanns J., Duchemin B., Staiger M.P., Medronho B. (2015). From Cellulose Dissolution and Regeneration to Added Value Applications—Synergism Between Molecular Understanding and Material Development. Cellulose—Fundamental Aspects and Current Trends.

[21] Zhang Y.H.P., Lynd L.R. (2005). Determination of the number-average degree of polymerization of cellodextrins and cellulose with application to enzymatic hydrolysis. Biomacromolecules.

[22] Cai J., Zhang L., Liu S., Liu Y., Xu X., Chen X., Chu B., Guo X., Xu J., Cheng H. (2008). Dynamic self-assembly induced rapid dissolution of cellulose at low temperatures. Macromolecules.

[23] Medronho B., Lindman B. (2015). Brief overview on cellulose dissolution/regeneration interactions and mechanisms. Adv. Colloid Interface Sci..

[24] Davidson G.F. (1934). 12—The dissolution of chemically modified cotton cellulose in alkaline solutions: Part I—In solutions of sodium hydroxide, particularly at temperatures below the normal. J. Text. Inst. Trans..

[25] Egal M., Budtova T., Navard P. (2007). Structure of aqueous solutions of microcrystalline cellulose/sodium hydroxide below 0 °C and the limit of cellulose dissolution. Biomacromolecules.

[26] Yang Q., Qi H., Lue A., Hu K., Cheng G., Zhang L. (2011). Role of sodium zincate on cellulose dissolution in NaOH/urea aqueous solution at low temperature. Carbohydr. Polym..

[27] Kihlman M., Medronho B.F., Romano A.L., Germgård U., Lindman B. (2013). Cellulose dissolution in an alkali based solvent: Influence of additives and pretreatments. J. Braz. Chem. Soc..

[28] Liu W., Budtova T., Navard P. (2011). Influence of ZnO on the properties of dilute and semi-dilute cellulose-NaOH-water solutions. Cellulose.

[29] Wang Y., Deng Y. (2009). The kinetics of cellulose dissolution in sodium hydroxide solution at low temperatures. Biotechnol. Bioeng..

[30] Bregado J.L., Tavares F.W., Secchi A.R., Segtovich I.S.V. (2021). Molecular dynamics of dissolution of a 36-chain cellulose Iβ microfibril at different temperatures above the critical pressure of water. J. Mol. Liq..

[31] Parthasarathi R., Balamurugan K., Shi J., Subramanian V., Simmons B.A., Singh S. (2015). Theoretical Insights into the Role of Water in the Dissolution of Cellulose Using IL/Water Mixed Solvent Systems. J. Phys. Chem. B.

[32] Gomes T.C., Skaf M.S. (2012). Cellulose–builder: A toolkit for building crystalline structures of cellulose. J. Comput. Chem..

[33] Kevin J.B. Scalable Algorithms for Molecular Dynamics Simulations on Commodity Clusters. Proceedings of the 2006 ACM/IEEE Conference on Supercomputing.

[34] Kang Y., Wang F., Zhang Z., Zhou J. (2021). Dissolution and interaction of cellulose carbamate in naoh/zno aqueous solutions. Polymers.

[35] Manna B., Ghosh A. (2019). Dissolution of cellulose in ionic liquid and water mixtures as revealed by molecular dynamics simulations. J. Biomol. Struct. Dyn..

[36] Chen Y., Jiang Y., Wan J., Wu Q., Wei Z., Ma Y. (2018). Effects of wet-pressing induced fiber hornification on hydrogen bonds of cellulose and on properties of eucalyptus paper sheets. Holzforschung.

[37] Liu H., Sale K.L., Simmons B.A., Singh S. (2011). Molecular dynamics study of polysaccharides in binary solvent mixtures of an ionic liquid and water. J. Phys. Chem. B.

[38] Tian G., Du H., Yuan Q. (2021). The effects of benzene on the structure and properties of triethylamine hydrochloride/chloroaluminate. Crystals.

[39] Gupta K.M., Hu Z., Jiang J. (2013). Molecular insight into cellulose regeneration from a cellulose/ionic liquid mixture: Effects of water concentration and temperature. RSC Adv..

